# Serum and Dietary Folate and Vitamin B_12_ Levels Account for Differences in Cellular Aging: Evidence Based on Telomere Findings in 5581 U.S. Adults

**DOI:** 10.1155/2019/4358717

**Published:** 2019-10-07

**Authors:** Larry A. Tucker

**Affiliations:** College of Life Sciences, Brigham Young University, Provo, Utah 84602, USA

## Abstract

Folate and vitamin B_12_ are essential for a variety of metabolic processes. Both micronutrients have been shown to reduce oxidative stress significantly. The present cross-sectional investigation evaluated the association between serum and dietary folate and vitamin B_12_ levels and leukocyte telomere length, an index of cellular aging influenced by oxidative stress. The study included 5581 adults from the National Health and Nutrition Examination Survey (NHANES). Because participants were randomly selected, results are generalizable to all civilian, noninstitutionalized U.S. adults. A blood draw provided DNA and serum folate and B_12_ information. The quantitative polymerase chain reaction method was used to measure telomere length. The Bio-Rad Quantaphase II folate and vitamin B_12_ radioassay kit was used to quantify levels of folate and vitamin B_12_. Dietary folate and vitamin B_12_ were assessed using a multipass 24 h recall. In some models, age, race, smoking pack-years, alcohol use, body mass index, total physical activity, hours fasted before the blood draw, and diabetes status were employed as covariates to minimize their influence. Findings showed that for each additional year of chronological age, telomeres were 15.6 base pairs shorter, on average (*F* = 378.8, *p* < 0.0001). Men had shorter telomeres than women after adjusting for all the covariates (*F* = 6.8, *p* = 0.0146). Serum (*F* = 10.5, *p* = 0.0030) and dietary (*F* = 5.0, *p* = 0.0325) folate concentrations were each linearly related to telomere length in women, but not in men, after controlling for age and race. Serum vitamin B_12_ and telomere length had a nonsignificant, inverse relationship in women, with age and race controlled (*F* = 2.8, *p* = 0.1056), but no relation in men. Dietary vitamin B_12_ was linearly related to telomere length in women, after adjusting for age and race (*F* = 4.3, *p* = 0.0468), but not in men. Overall, evidence indicates that folate and vitamin B_12_ levels, especially folate, account for meaningful differences in cell aging in women, but not in men.

## 1. Introduction

A good measure of DNA stability, cellular integrity, and biologic aging is the length of telomeres. Telomeres are nucleoprotein structures that cap the ends of chromosomes. Because the ends of telomeres cannot be completely replicated when cells divide, telomeric DNA is used to safeguard genetic code, so it is not lost from the ends of chromosomes. Telomeres naturally shorten during each cell division. The loss of telomere base pairs is sometimes called “the end replication problem,” but other factors can cause telomere shortening. Eventually, telomeres become critically short and senescence and cell death occur. Consequently, morbidity and mortality associated with several disorders are higher in adults with shorter telomeres [1–3].

Oxidative stress plays a major role in the length of telomeres and in cell aging [4, 5]. Telomeres are highly sensitive to injury caused by oxidative stress because of their significant guanine content [6]. Additionally, telomeres tend to be affected by the accumulation of ROS-induced DNA-strand breaks [6].

A number of dietary factors can influence oxidative stress levels in the body. For example, research indicates that whole foods, such as fruits and vegetables, are significantly related to lower levels of oxidative stress [7–9]. Similarly, nuts and seeds seem to diminish inflammation and oxidative stress as well [10–12]. Furthermore, as consumption of vitamin D increases, markers of oxidative damage tend to decrease [13–16].

Several investigations show that dietary factors that reduce inflammation and oxidative stress also tend to influence cellular aging favorably. There are a number of examples. Studies by Lian et al. [17] and also by Gong et al. [18] provide evidence of significant associations between vegetable intake and reduced biologic aging, indexed by telomere length. Research by García-Calzón et al. indicates that total antioxidant capacity of the diet is connected with diminished cell aging [19]. Likewise, higher vitamin D concentrations are associated with longer telomeres, according to Richards et al. [20], Liu et al. [21], and Pusceddu et al. [22].

On the other hand, a 2018 meta-analysis by Pérez et al. that included five randomized controlled trials covering nine different diets investigated the effects of diet on biologic aging. Collectively, the meta-analysis showed no effect of diet on telomere length [23]. Similarly, studying 2509 apparently healthy middle-aged men and women, De Meyer et al. found no association between overall dietary characteristics, including a dietary inflammatory index, and telomere length [24]. In a comprehensive 2016 review paper focusing on diet and telomere length, Freitas-Simoes et al. cited a number of investigations showing that some dietary factors are related to reduced biologic aging, but the paper also discussed several studies presenting evidence that diet is not linked to improved cellular aging and longer telomeres, even when foods containing significant antioxidants are consumed [25].

Folate and vitamin B_12_ are micronutrients required for essential metabolic functions [26]. The metabolism of both vitamins is closely intertwined. Deficiencies of folate and vitamin B_12_ are common clinical conditions with significant subclinical and clinical health consequences. Several studies show that individuals with low levels of these micronutrients tend to have elevated markers of oxidative stress [27–30].

Because folate and vitamin B_12_ are major players in the control of oxidative stress [27–30], and they also influence nucleotide synthesis and DNA stability [26, 31], these micronutrients have the potential to affect telomere structure and function and biologic aging. To date, the association between folate and vitamin B_12_ and telomere length has been studied directly only a few times and never in a U.S. national sample [32–37]. Moreover, findings have been inconsistent.

Hence, the purpose of the present investigation was to evaluate the relationship between folate and vitamin B_12_ levels, derived from the blood and also from the diet, on telomere length in 5581 women and men, representative of the U.S. adult population. Another objective was to determine the effect of age, sex, race, hours of fasting, smoking, alcohol use, body mass index, total physical activity, diabetes status, and pregnancy status, on the vitamin and cellular aging relationships.

## 2. Materials and Methods

### 2.1. Sample

NHANES, also known as the National Health and Nutrition Examination Survey, is an ongoing assessment of the health and lifestyles of women, men, and children living in the United States. All NHANES data are cross-sectional. NHANES randomly samples individuals using a multilevel design so that external validity is strong and results can be generalized to all civilian, noninstitutionalized people in the U.S.

Although NHANES has been collecting national data for many decades, only two 2-year survey cycles include information on telomere length, 1999–2000 and 2001–2002. The public did not have access to telomere data until November 2014. All NHANES data sets are free and available online to the general public [38].

Collection of blood samples by NHANES allowing access to the DNA of individuals was delimited to participants who were ≥20 years old. A sample of 10,291 men and women was eligible and 76% (7827) agreed to give a sample and actually provided a useable DNA sample. To ensure absolute confidentiality, all individuals who were age ≥85 were given the age of 85 by NHANES. Therefore, men and women age 85 or older were omitted from the sample used in this study.

Women and men were required to have complete data to be included in the investigation. A total of 5581 adults (2648 men and 2933 women) had data for all the variables used in this study. The National Center for Health Statistics Ethics Review Board sanctioned collection of the NHANES data and written informed consent was acquired from each subject [39].

### 2.2. Measures

Leukocyte telomere length was the outcome measure of the present investigation. Serum and dietary folate and vitamin B_12_ concentrations were the exposure variables. Covariates included age, race, hours of fasting before the blood draw, body mass index (BMI), pack-years of smoking, total MET-minutes of physical activity, alcohol use, diabetes status, and pregnancy status (for women). Sex was controlled by stratifying the sample, focusing on women and men separately.

#### 2.2.1. Telomere Length

The techniques utilized to calculate telomere length have been reported by NHANES in detail [40]. DNA was extracted from blood samples and stored at −80°C and then shipped to the Blackburn Laboratory at the University of California, San Francisco. At the lab, leucocyte telomere length was assessed by employing the quantitative polymerase chain reaction method. Samples were compared to standard reference DNA (T/S ratio) [40]. Five 96-well quality control plates, representing 5% of the complete set, were used. The investigators were blinded regarding the duplicate samples.

According to NHANES [40], the DNA samples were assayed using duplicate wells. Each sample was assayed on three different days and three times on each day. Three assay plates were analyzed together, and no two plates were grouped more than one time together. The assay plates had eight control DNA samples and 96 control wells. When there was eight or more invalid control wells associated with an assay run, the wells were removed from further analysis. This occurred less than 1% of the time. To normalize between-run variability, control DNA values were employed. Assay runs were excluded from further analysis when more than four DNA values fell outside of 2.5 standard deviations from the mean. This occurred in less than 6% of the runs. Evaluation showed that the coefficient of variation was 6.5% for the telomere measurements [40]. The formula 3274 + 2413 × (T/S) was employed to convert the mean T/S ratio values to base pairs.

#### 2.2.2. Folate and Vitamin B_12_

NHANES mobile examination centers (MECs) were used for the collection of blood specimens. Serum concentrations of folate and vitamin B_12_ were evaluated using the Bio-Rad Laboratories Quantaphase II folate/vitamin B_12_ radioassay kit [41].

Exclusion criteria included hemophiliacs, individuals who had received chemotherapy during the previous 4 weeks, and participants with rashes, gauze dressings, casts, edema, open sores or wounds, etc. The laboratory staff included certified medical technologists and phlebotomists. Members of the laboratory staff each completed comprehensive training before working in the MEC.

To measure the dietary levels of folate and vitamin B_12_, a 24 h dietary recall interview was conducted by NHANES by employing a computer-based system [42]. The dietary assessment was utilized to gather comprehensive information about all foods, beverages, and supplements consumed during the 24-hour period prior to the interview (midnight to midnight). During the dietary assessment, a multipass format was employed. Each interviewer was a college graduate and was formally educated in the area of food and nutrition. Interviews were handled in a private environment in an NHANES mobile examination center (MEC). Each interviewer was bilingual. The NHANES computer-assisted program provided a standardized interview format, and interviewers used scripts built into the system [42].

#### 2.2.3. Covariates

A number of covariates were used to control for differences in a variety of potential mediating factors. The demographic covariates were age and race, and subjects were stratified according to sex. Five categories were used by NHANES to distinguish among races and ethnicities: non-Hispanic White, non-Hispanic Black, Mexican American, Other race or Multiracial, and Other Hispanic. Several additional variables were measured to allow differences to be controlled, including hours of fasting before the blood draw, alcohol use, body mass index, smoking pack-years, total METs of physical activity, diabetes status, and, for females, pregnancy status.

#### 2.2.4. Hours of Fasting

Food intake can influence serum concentrations of vitamins, so hours of fasting prior to the blood draw were measured by NHANES. To encourage an overnight fast, blood draws were administered in the morning. Average fasting time was 10.0 ± 4.7 hours. About 90% of the participants fasted at least 5.0 hours and about 10% fasted 15.5 hours or longer. Hours of fasting was employed as a covariate because participants varied in the amount of time they fasted before the blood draw.

#### 2.2.5. Body Mass Index (BMI)

BMI was indexed using the formula weight (kg) ÷ height (m^2^), which allows the weight of adults to be compared, independent of height. Standard cut points were used to develop BMI categories for use in the present study: underweight (<18.5), normal weight (≥18.5 and <25.0), overweight (≥25.0 and <30.0), obese (≥30.0), or missing [43].

#### 2.2.6. Smoking

Long-term exposure to the effects of smoking was indexed by quantifying pack-years of cigarette smoking. Pack-years was estimated as the number of years the individual reported smoking times the average number of cigarettes reported smoking per day, divided by 20.

#### 2.2.7. Physical Activity

Participation in leisure time physical activity was used as a covariate. A total of 48 different physical activities were listed, and subjects identified the ones they participated in, if any, in the past month. Subjects were also required to identify if the intensity of the activity was moderate or vigorous, using NHANES explanations to guide them. Additionally, subjects reported the average duration of the activity. NHANES did not count participation in an activity if it lasted less than 10 minutes. A MET score was calculated for each individual, and total MET-minutes per week were estimated for each participant using the compendium of physical activities [44]. The total MET-minute value, treated as a continuous variable, was used to represent the physical activity level of each individual in the study.

#### 2.2.8. Alcohol Use

Alcohol use was also employed as a covariate. Subjects were separated into three categories based on their self-reported alcohol drinking habits: heavy drinkers, moderate drinkers, and abstainers. Men who indicated they drank three or more alcoholic drinks per day over the past 12 months were labeled heavy drinkers, whereas women were labeled heavy drinkers if they consumed two or more alcoholic beverages per day. Men who drank more than zero and less than three alcoholic beverages per day were defined as moderate drinkers, whereas women were moderate drinkers if they drank more than zero but less than two drinks per day over the past 12 months. Adults who indicated they did not drink alcohol in the past 12 months were labeled abstainers.

#### 2.2.9. Diabetes Status

Participants who had fasting blood glucose levels of 126 mg/dL or higher were defined as diabetic. Additionally, adults who indicated they had been told by their physician that they were diabetic, or they were taking insulin or a prescription medication for their diabetes, were considered diabetic. The diabetes status covariate was treated as a dichotomous variable: diabetic or normal.

#### 2.2.10. Pregnancy Status

Female participants reported whether they were pregnant. Pregnancy status was treated as a dichotomous covariate: pregnant or not pregnant.

### 2.3. Statistical Analysis

NHANES used a multilevel random sampling strategy to produce findings generalizable to the noninstitutionalized, civilian, adult population of the United States. In general, counties within the United States were randomly selected initially, then roads, then houses, and then individuals. Individual sampling weights, which were assigned to each participant by NHANES, were included in each analysis resulting in unbiased national estimates. SAS SurveyFreq was employed to produce weighted frequencies to describe the categorical data. SAS SurveyMeans was used to generate weighted means (±SE) to describe continuous variables.

Serum and dietary levels of folate and vitamin B_12_ were the exposure variables. Their distributions deviated from normal, so they were log-transformed before inclusion in the analyses. Leukocyte telomere length was the outcome variable.

The SAS SurveyReg procedure, taking into account strata, clusters, and individual sampling weights, was utilized to calculate the extent of the linear associations between folate and vitamin B_12_ and telomere length, including the linear relationships between serum and dietary levels of the vitamins. In the present investigation, with age and race controlled, women had significantly longer telomeres than men. Women and men also differed in their levels of folate and vitamin B_12_. Hence, the relationships between folate and vitamin B_12_ and telomere length were evaluated separately for women and men.

Although a sample of more than 5500 individuals was used in the present investigation, statistical power was not extreme. In particular, the multistage, nested sampling strategy of NHANES produced 29 degrees of freedom (df) in the denominator for all analyses, including those delimited to women only and men only. The 29 degrees of freedom were derived by subtracting the 28 NHANES strata from the 57 NHANES clusters.

Differences in the demographic variables (age and race) were controlled statistically using partial correlation and the SAS SurveyReg procedure. Adjustments were also made for additional potential mediating variables using partial correlation, including the number of hours fasted before the blood draw, BMI, smoking, physical activity, alcohol use, diabetes status, and pregnancy status (for females) to test their effect on the vitamin and telomere length associations. Sex was controlled by stratifying the sample into two categories, women and men.

Alpha was set at the 0.05 level and all *p* values were two-sided. SAS version 9.4 was used to conduct the statistical analyses (SAS Institute, Inc., Cary, NC, USA).

## 3. Results

Individual sample weights were used to produce findings generalizable to the noninstitutionalized, civilian adult population of the United States. Using the total sample, average age (±SE) was 46.4 ± 0.4 years and mean telomere length was 5833 ± 40 base pairs. Average serum levels of folate and vitamin B_12_ were 14.4 ± 0.2 ng/mL and 509.5 ± 7.1 pg/mL, respectively. Mean dietary folate and vitamin B_12_ intakes were 408.5 ± 8.4 mcg per day and 5.3 ± 0.2 mcg per day, respectively.

The relationship between serum folate and dietary folate was 0.20 (*F* = 52.7, *R*^2^ = 0.042, *p* < 0.0001) in women and 0.16 (*F* = 29.2, *R*^2^ = 0.026, *p* < 0.0001) in men. Similarly, the association between serum vitamin B_12_ and dietary B_12_ was 0.11 (*F* = 22.9, *R*^2^ = 0.011, *p* < 0.0001) in women and 0.13 (*F* = 37.4, *R*^2^ = 0.017, *p* < 0.0001) in men.

Median levels of the folate and vitamin B_12_ variables are displayed in Table 1. Overall, Table 1 shows the weighted percentiles (±SE), including the 5^th^, 25^th^, 50^th^, 75^th^, and 95^th^ percentiles, for the exposure and outcome variables of the present investigation. The results in Table 1 are based on analyses using strata, clusters, and individual sample weights, and therefore, the findings represent the U.S. adult population.

### 3.1. Age and Telomeres

The association between chronological age and telomere length was linear and inverse. Telomeres were 15.6 base pairs shorter for each additional year of chronological age within the sample (*F* = 378.8, *p* < 0.0001). The Pearson correlation between age and telomere length was -0.42 (*p* < 0.0001).

### 3.2. Men, Women, and Telomeres

In the present sample, men and women differed significantly in the length of their telomeres. Specifically, after adjusting for differences in age and race, average telomere length was significantly shorter in men than women, as shown in Table 2. After controlling for differences in all the covariates, the mean difference between women and men in telomere length remained statistically significant (Table 2).

### 3.3. Serum Folate and Telomere Length

As displayed in Table 3, regression analysis results showed that serum concentrations of folate were linearly related to telomere length in women, after adjusting for differences in the demographic covariates (*F* = 10.5, *p* = 0.0030). For each 10% increase in serum folate, telomeres were 9.57 base pairs longer, on average. In men, the association between serum folate and telomere length was positive, but not significant, after controlling for the demographic covariates (*F* = 2.9, *p* = 0.0972). In women, with all the covariates controlled (Model 2), for each 10% increase in serum folate, telomeres were 8.75 base pairs longer, on average (*F* = 8.7, *p* = 0.0062). In men, with all the covariates controlled (Model 2), serum folate and telomere length were not related (*F* = 1.9, *p* = 0.1838).

### 3.4. Dietary Folate and Telomere Length

As displayed in Table 3, dietary folate levels were linearly related to telomere length in women, but not in men. In women, both Model 1 and Model 2 were significant. After adjusting for all the potential mediating variables, for each 10% increase in dietary folate intake, telomeres were 5.98 base pairs longer, on average (*F* = 4.3, *p* = 0.0479).

### 3.5. Serum Vitamin B_12_ and Telomere Length

In women, after adjusting for differences in age, race, smoking, alcohol use, BMI, total physical activity, hours fasted, diabetes status, and pregnancy status, for each 10% increase in serum vitamin B_12_, telomeres were 5.59 base pairs longer, on average (*F* = 3.1, *p* = 0.0867). The association was also not significant in men (*F* = 0.4, *p* = 0.5227).

### 3.6. Dietary Vitamin B_12_ and Telomere Length

After controlling for age and race, vitamin B_12_ intake was linearly and positively related to telomere length in women (*F* = 4.3, *p* = 0.0468). The association was not significant in men. In women, Model 1 revealed that for each 10% increase in dietary B_12_ reported, telomeres were 2.12 base pairs longer. However, when all the covariates were controlled, the relationship was weakened to the point of nonsignificance (*F* = 2.8, *p* = 0.1080).

## 4. Discussion

The primary purpose of this investigation was to determine the relationship between serum and dietary concentrations of folate and vitamin B_12_ and leukocyte telomere length, an objective marker of cellular aging, within a large, random, national sample of U.S. women and men, ages 20-84. Findings differed between women and men (Table 2). In women, telomeres were consistently longer, signifying less biologic aging, as levels of folate increased (Table 3). The relationships were meaningful whether or not folate was measured in the blood or from the diet. However, this was not the case in men. The associations between serum and dietary vitamin B_12_ levels and telomere length in women were less consistent, however, and none of the associations were significant in men (Table 3).

As expected, telomeres were progressively shorter for each additional year of chronological age. The relationship between age and telomere length was inverse, linear, and substantial. Specifically, for each additional year of age, telomeres were 15.6 base pairs shorter, on average. Interpretation of these results is straightforward. For example, over any 10-year span of chronological age, the average difference in telomere length was roughly 156 base pairs in the present sample (10 × 15.6 = 156). If two groups in this sample differed in age by 3 years, the mean telomere length of the older group would be roughly 47 base pairs shorter than the younger group, on average (15.6 × 3 = 46.8).

Given the robust, linear associations between folate, serum and dietary, and telomere length in women, it follows that cellular aging was less in women who had high levels of folate compared to their counterparts. Specifically, with all the covariates controlled, for each 10% increase in serum folate, telomeres were 8.75 base pairs longer, on average (Table 3). Interpretation of these findings using an example would be as follows. If two groups of women differed by 50% in their serum folate levels, then the cellular aging difference between the groups would be approximately 43.75 telomere base pairs (8.75 × 5 = 43.75) or 2.8 years of biological aging (43.75 ÷ 15.6 = 2.8). If one group had double the serum folate levels compared to the other, similar to the difference between the 25^th^ and 75^th^ percentiles (Table 1), then the average cellular aging difference would be roughly 5.6 years (8.75 × 10 = 87.5 ÷ 15.6 = 5.6).

The estimated cellular aging difference based on dietary folate levels in women was less than those based on serum concentrations. Multiple factors likely account for this finding. Dietary estimates were based on a self-reported dietary recall compared to the blood folate values, which were objectively measured. In short, serum concentrations represent the real-time status of folate in the body. Serum levels reflect the net outcome of bioavailability and metabolism of folate. However, as indicated in a recent review by Thurnham and Northrop-Clewes, nutrient biomarkers are not foolproof. They can be thrown off by infection and inflammation. Moreover, in another review, Elmadfa and Meyer suggest that nutrient biomarkers and dietary assessments complement each other. Both have their strengths and weaknesses. Consequently, both were used in the present study.

Although the relationships between folate and vitamin B_12_ and telomere length did not reach statistical significance in men, most of the associations were meaningful in women. Women in the highest quartile of dietary folate reported more than twice the folate intake compared to those in the lowest quartile. Given the relationships in women tended to be linear, estimates of the cellular aging advantages were substantial. Few other dietary factors are associated with greater aging benefits. For example, a recent publication focused on the consumption of nuts and seeds and telomere length. For each 10% increment in energy derived from nuts and seeds, biologic aging decreased by 3.3 years, on average [45]. In another study based on dietary fiber and telomere length, for each 10 gram increase per day in fiber intake, cell aging was 4.3 years less, on average [46]. Given these comparisons, high levels of folate accounted for cellular aging benefits that apparently exceed a 10% increase in consumption of nuts and seeds or a 10-gram increase per day in fiber intake, particularly in U.S. women.

The U.S. Recommended Daily Allowance (RDA) for folate in the U.S. is 400 mcg for adults. According to the dietary findings in the present national sample, median (±SE) levels were low in women and very close to the RDA in men. Median intake was 311.9 ± 8.1 mcg in women, 22% below the RDA, on average. Median intake in men was 398.0 ± 11.4 mcg, nearly identical to the RDA. In the present sample, 68% of women in the U.S. consumed less than the RDA for folate and 50% of men fell short of the RDA. Given shorter telomeres and increased cellular aging were more common in women who consumed lower levels of folate, there is clearly room for improvement.

U.S. men and women seem to be doing better regarding vitamin B_12_ consumption. The RDA for vitamin B_12_ in the U.S. is 2.4 mcg for adults. Median (±SE) intake in the present sample was 3.2 ± 0.1 mcg in women and 4.7 ± 0.1 mcg in men. Approximately 37% of women and 20% of men were below the RDA for vitamin B_12_ intake in this sample.

Mean dietary folate and vitamin B_12_ levels were higher in men than in women, and U.S. women were much more likely to have intakes below the RDA for folate and vitamin B_12_. This may partially account for the demonstrated stronger relationships between folate and vitamin B_12_ and telomere length in women in the present study. In short, women could be more susceptible to DNA damage resulting from lower dietary levels of folate or vitamin B_12_. The obvious difference in the link between folate and vitamin B_12_ and telomere length between women and men warrants further research.

Although U.S. women reported substantially lower dietary intakes of folate and vitamin B_12_ than men, women had higher serum concentrations of folate than men. Regarding serum levels of vitamin B_12_, men and women had similar median levels, but women had higher highs and lower lows, more extreme levels. How can this be? Specifically, how could U.S. women report lower dietary consumption of folate yet have higher serum concentrations of folate? Although there are multiple possibilities, one reason stands out: underreporting. When reporting dietary intake, both men and women tend to report eating less food than they actually consume [47]. Women, however, tend to underreport substantially more than men [47]. This would account for the lower dietary intakes of folate and vitamin B_12_ reported by women, but the higher serum folate levels and similar vitamin B_12_ levels in U.S. women compared to men.

The correlation between dietary folate and serum concentrations of folate was stronger in women than in men. This would suggest that dietary intake of folate, at least self-reported consumption, plays a more significant role in serum concentrations among women than men. The correlation was weak between dietary vitamin B_12_ and serum levels of B_12_ in both men and women, indicating that there is not much overlap between the two.

A number of investigations indicate that diets with significant antioxidant levels tend to promote cell longevity, including vegetables [17, 18], vitamin D [20–22], low glycemic loads [19], polyunsaturated fatty acids [19], and foods scoring high collectively on an antioxidant capacity index [19]. However, the relationship is not automatic. A recent meta-analysis by Pérez et al., focusing on nine different diets, indicated that diet has no effect on telomere length [23]. Likewise, others have observed no relationship between a dietary inflammatory index and telomere length in over 2500 adults [24]. In a recent study that focused on alpha- and gamma-tocopherol and telomere length, gamma-tocopherol was actually inversely related to telomere length [48]. In general, it appears that some macro- and micronutrients are associated with reduced cellular aging and some are not, as shown in the literature review by Freitas-Simoes et al. [25].

Dietary folate and vitamin B_12_ both seem to possess antioxidant qualities. Consequently, each has the potential to prevent the shortening of telomeres and the reduction of premature biologic aging. For example, Cagnacci et al. [27] conducted a double-blind randomized controlled trial with 30 apparently healthy postmenopausal women. Treatment was 15 mg per day of 5-methyltetrahydrofolate or placebo administered for three weeks. Results showed a clear and significant reduction in oxidative stress, indexed using several measures. Misra et al. [49] studied 51 individuals with vitamin B_12_ deficiency and 53 adults without. Findings showed that those with low B_12_ levels had significantly higher levels of several measures of oxidative stress than their counterparts. Joshi et al. [50] examined the free radical scavenging properties and possible antioxidant activity of folic acid. In the reaction of thiyl radicals with folic acid, it was shown that folic acid not only scavenged thiyl radicals but also repaired the thiyls at the physiological pH. The authors also observed a significant inhibition property in microsomal lipid peroxidation associated with folic acid. Finally, in a review of the literature by van de Lagemaat et al. [51], 15 investigations were evaluated regarding the effect of vitamin B_12_ intake on oxidative stress. The authors established that lower concentrations of vitamin B_12_ are consistently predictive of higher levels of prooxidants and lower levels of antioxidants. The authors hesitated to conclude that vitamin B_12_ reduces oxidative stress causally, however, because most of the studies they reviewed that involved humans were not controlled investigations.

To date, few investigations have studied directly the relationship between folate and vitamin B_12_ levels and telomere length, and none have used a U.S. national sample. Moreover, results have been inconsistent. For example, using a subsample of 1715 women from the Nurses' Health Study, Liu et al. concluded that plasma and also dietary concentrations of folate and vitamin B_12_ were not related to telomere length [32]. Likewise, in a study by Bull et al., the association between plasma folate and vitamin B_12_ and telomere length were examined in Australian men and women. A total of 43 young adults and 47 older adults were evaluated. Dietary folate and vitamin B_12_ levels were not related to plasma concentrations of the vitamins [33]. Also, Shin and Baik studied 798 women and men aged 55-79 years old and the association between serum folate, vitamin B_12_, and leukocyte telomere length, and other variables. They found no relationship between folate, B_12_, and telomere length [34].

On the other hand, Richards et al. studied a sample of 1319 healthy adults from the United Kingdom. A significant relationship was reported between serum folate levels and telomere length, after adjusting for differences in age [35]. The association between vitamin B_12_ and telomere length was not examined. The sample was approximately 92% women.

An investigation by Paul et al. included 195 men from Italy. The researchers found a nonlinear relationship between plasma concentrations of folate and telomere length. When folate levels were above the median, the relationship with telomere length was positive, but when folate levels were below the median, the association was inverse [36]. Although vitamin B_12_ concentrations were measured, the relationship between B_12_ and telomere length was not evaluated.

Lastly, using 1044 older men and women from the Framingham Offspring Study (mean age: 59 years), the scientists reported that the relationship was inverse and linear when delimited to the 2^nd^–5^th^ quintiles of plasma folate levels. However, the association was not significant when the 1^st^ quintile was included. Findings were not reported for men and women separately, and the association between vitamin B_12_ and telomere length was not reported [37].

In the present study, as levels of folate and vitamin B_12_ decreased in women, telomeres were progressively shorter. Multiple mechanisms could account for this finding. For example, oxidative stress plays a key role in the length of telomeres and in cell aging [4, 5]. Oxidative stress tends to cause telomeres to shorten [6]. Numerous studies by von Zglinicki et al. [5, 52–55] and others reviewed by Houben et al. [4], using a variety of animal species, support this relationship. Since adults with lower levels of folate and/or vitamin B_12_ are inclined to have elevated oxidative stress [27–30, 50, 51, 56], it follows that these individuals would also tend to have shorter telomeres [4–6].

The same mechanism can also be considered using a reversed perspective. Specifically, the association between oxidative stress and lower concentrations of serum folate and vitamin B_12_ could be a result of folate and B_12_ serving as antioxidants. Once oxidized, folate and B_12_ are no longer biologically active. In short, rather than low folate or B_12_ concentrations leading to oxidative stress, oxidative stress could lead to lower levels of serum folate and B_12_. Since women may consume significantly less folate and vitamin B_12_ than men, sometimes, these micronutrients may not be maintained at adequate concentrations in women. This could explain why low levels of folate and vitamin B12 were linked to short telomeres in women but not in men.

Other factors accounting for the folate, vitamin B_12_, and telomere associations are also possible. From a molecular perspective, Moore et al. offer multiple related mechanisms to explain the association between folate concentrations and telomere length. They indicate that low folate levels could lead to shorter telomeres by removal of increased uracil in the telomere hexamer repeat (TTAGGG), which has been shown to produce DNA breaks and abasic sites (AP sites). Additionally, the association could be partly due to incompetent binding of shelterin proteins to telomeric DNA due to decreased affinity with uracil and AP sites because of uracil repairs in the telomere repeat [57].

The relationship between folate and vitamin B_12_ and biologic aging was consistent and meaningful in women, but not in men. Given oxidative stress is a key factor associated with shorter telomeres, and low levels of folate and vitamin B_12_ each tend to be linked to higher levels of oxidative stress, it follows that the relationship between folate and vitamin B_12_ and telomere length would be expected to be stronger in men. Undoubtedly, other factors are involved in the folate, B_12_, and telomere associations, especially in men.

The current study had several limitations. First, causal conclusions are not valid because the investigation used a cross-sectional design. Additionally, the significant findings could be partly a result of residual confounding. In other words, high levels of folate and vitamin B_12_ could be biomarkers of a healthy lifestyle, which could be the underlying factor accounting for the longer telomeres found in the women of this investigation. Similarly, although multiple potential confounders were controlled in the present study, there could be other factors that might explain the positive association between folate and B_12_ and telomere length.

The present investigation also had a number of strengths and novel characteristics: (1) Participants were randomly selected. Hence, the results are generalizable to all noninstitutionalized, civilian adults of the United States. (2) The random sample included 5581 adults. As a result, findings are stable. (3) Chronological age was strongly related to telomere length, attesting to the precision and validity of the telomere measurement protocol by NHANES and the Blackburn Laboratory at the University of California, San Francisco. (4) Using telomere base pairs, biological aging differences were presented in chronological years, facilitating a straightforward understanding of the strength of the relationship between folate and vitamin B_12_ and cell aging. (5) The sample incorporated all races and adults 20-84 years old. (6) A total of 10 potential confounding variables were controlled statistically, providing evidence that the key results of the present study are robust. (7) Both serum concentrations and dietary levels of folate and vitamin B_12_ were studied.

## 5. Conclusions

In conclusion, results from a nationally representative sample of U.S. adults indicate that cellular aging tends to increase linearly as levels of folate and vitamin B_12_ decrease in women, but not in men. The observed relationship in women could be a function of low concentrations of folate and vitamin B_12_ resulting in higher oxidative stress levels, leading to accelerated cell aging and shorter telomeres. The reverse is also logical—oxidative stress leading to lower levels of folate and vitamin B_12_, resulting in telomere attrition, particularly in women. The relationship could also be due to these vitamins influencing the structure and function of DNA and the epigenetic regulation of telomeres through DNA methylation. Whatever the mechanism, it appears that the micronutrients folate and vitamin B_12_, especially the former, play important roles in the biologic aging of women. The role of these vitamins in connection with telomere length and aging in men is less apparent. Clearly, additional research focusing on folate and vitamin B_12_ and biologic aging is warranted, particularly randomized controlled trials, so that causality can be deciphered.

## Figures and Tables

**Table 1:** Percentiles for the exposure and outcome variables representing U.S. women and men.

Variable	Percentile (±SE)				
	5th	25th	50th	75^th^	95th
*Serum folate (ng/mL)*					
Women (*n* = 2933)	5.7 ± 0.1	9.4 ± 0.1	13.1 ± 0.2	18.6 ± 0.3	31.1 ± 0.9
Men (*n* = 2648)	5.3 ± 0.2	8.5 ± 0.2	12.0 ± 0.2	16.1 ± 0.3	25.5 ± 0.7
Combined (*n* = 5581)	5.5 ± 0.1	9.0 ± 0.2	12.4 ± 0.2	17.5 ± 0.3	28.8 ± 0.7
*Serum vitamin B_12_ (pg/mL)*					
Women (*n* = 2933)	217.1 ± 5.2	335.7 ± 4.3	449.3 ± 5.8	598.8 ± 6.9	968.4 ± 27.7
Men (*n* = 2648)	235.5 ± 4.6	357.6 ± 6.1	452.6 ± 6.6	588.3 ± 6.9	840.0 ± 19.3
Combined (*n* = 5581)	225.8 ± 3.6	344.8 ± 3.0	451.4 ± 4.4	594.2 ± 5.8	894.6 ± 23.0
*Dietary folate (mcg)*					
Women (*n* = 2928)	113.6 ± 5.6	214.8 ± 5.6	311.9 ± 8.1	438.9 ± 8.3	745.7 ± 19.5
Men (*n* = 2644)	135.1 ± 8.0	261.2 ± 6.2	398.0 ± 11.4	573.9 ± 13.8	1039 ± 44.5
Combined (*n* = 5572)	121.8 ± 4.3	236.5 ± 5.3	349.7 ± 8.4	502.0 ± 11.7	889.3 ± 36.8
*Dietary vitamin B_12_ (mcg)*					
Women (*n* = 2928)	0.5 ± 0.0	1.7 ± 0.1	3.2 ± 0.1	5.4 ± 0.1	10.5 ± 0.5
Men (*n* = 2644)	1.0 ± 0.1	2.7 ± 0.1	4.7 ± 0.1	7.8 ± 0.3	15.2 ± 0.6
Combined (*n* = 5572)	0.7 ± 0.1	2.1 ± 0.1	3.9 ± 0.1	6.4 ± 0.2	13.2 ± 0.7
*Telomere length (base pairs)*					
Women (*n* = 2933)	4951 ± 38	5402 ± 38	5759 ± 40	6177 ± 55	7061 ± 136
Men (*n* = 2648)	4960 ± 32	5358 ± 28	5730 ± 33	6155 ± 47	6975 ± 97
Combined (*n* = 5581)	4958 ± 33	5385 ± 31	5745 ± 34	6171 ± 48	7022 ± 99

Note: SE: standard error. Table values include person-level weighted adjustments based on the sampling methods of NHANES so that values represent those of the U.S. adult population.

**Table 2:** Mean differences in telomere length (base pairs) between U.S. men and women (*n* = 5581).

	Men	Women		
	Mean ± SE	Mean ± SE	*F*	*p*
Telomere length				
Model 1	5765 ± 35	5824 ± 40	4.6	0.0402
Model 2	5796 ± 38	5855 ± 41	6.8	0.0146

SE: standard error. Means have been adjusted for the covariates, as defined by each model. Model 1 included adjustment for differences in age and race. Model 2 included adjustment for differences in age and race, as well as pack-years of smoking, alcohol use, BMI, total physical activity, hours of fasting, and diabetes status. Mean differences between women and men for Models 1 and 2 are significant (*p* < 0.05).

**Table 3:** Relationship between serum and dietary levels of folate and vitamin B12 (per 10% increase) and telomere length (base pairs) in U.S. women and men.

	Telomere length (base pairs)			
Exposure variable	Regression coefficient	SE	*F*	*p*
*Women*				
Serum folate (ng/mL)				
Model 1	9.57	2.75	10.5	0.0030
Model 2	8.75	2.94	8.7	0.0062
Dietary folate (mcg)				
Model 1	5.41	2.41	5.0	0.0325
Model 2	5.98	2.89	4.3	0.0479
Serum vitamin B_12_ (pg/mL)				
Model 1	3.48	2.01	2.8	0.1056
Model 2	5.59	3.15	3.1	0.0867
Dietary vitamin B_12_ (mcg)				
Model 1	2.12	1.02	4.3	0.0468
Model 2	1.94	1.17	2.8	0.1080
*Men*				
Serum folate (ng/mL)				
Model 1	5.81	3.39	2.9	0.0972
Model 2	4.56	3.35	1.9	0.1838
Dietary folate (mcg)				
Model 1	2.27	2.86	0.6	0.4341
Model 2	0.71	2.99	0.1	0.8149
Serum vitamin B_12_ (pg/mL)				
Model 1	-1.92	3.58	0.3	0.5948
Model 2	-2.32	3.59	0.4	0.5227
Dietary vitamin B_12_ (mcg)				
Model 1	0.72	1.66	0.2	0.6665
Model 2	0.16	1.66	0.0	0.9239

SE: standard error. For Model 1, the covariates were age and race. For Model 2, in additional to age and race, the model was adjusted for differences in pack-years of smoking, alcohol use, BMI, total physical activity, hours fasted, diabetes status, and pregnancy status (for women). Interpretation of the regression coefficients is as follows for the first row (Model 1) regarding serum folate (ng/mL) in women: after adjusting for differences in age and race, for each 10% increase in serum folate, telomeres were 9.57 base pairs longer, on average.

## Data Availability

The data used to support the findings of this study were supplied online by the U.S. Centers for Disease Control and Prevention, as part of the National Health and Nutrition Examination Survey (NHANES). The NHANES data and related documentation are free to the public and available at https://wwwn.cdc.gov/nchs/nhanes/Default.aspx.
